# The impact of professional midwives and mentoring on the quality and availability of maternity care in government sub-district hospitals in Bangladesh: a mixed-methods observational study

**DOI:** 10.1186/s12884-022-05096-x

**Published:** 2022-11-08

**Authors:** Rondi Anderson, Anna Williams, Nicole Jess, Jonathan M. Read, Mark Limmer

**Affiliations:** 1 UNFPA, Dhaka, Bangladesh; 2 Centre for Health Inequalities Research, Division of Health Research, Faculty of Health and Medicine, Lancaster University, Lancaster, UK; 3 Data, Design + Writing, Oregon, USA; 4 Center for Statistical Training & Consulting, Measurement & Quantitative Methods, Michigan State University, Michigan, USA

**Keywords:** ICM standard midwives, Mentorship, Quality of care, Respectful maternity care, Bangladesh, Health system strengthening

## Abstract

**Background:**

This study compared government sub-district hospitals in Bangladesh without globally standard midwives, with those with recently introduced midwives, both with and without facility mentoring, to see if the introduction of midwives was associated with improved quality and availability of maternity care. In addition, it analysed the experiences of the newly deployed midwives and the maternity staff and managers that they joined.

**Methods:**

This was a mixed-methods observational study. The six busiest hospitals from three pre-existing groups of government sub-district hospitals were studied; those with no midwives, those with midwives, and those with midwives and mentoring. For the quantitative component, observations of facility readiness (*n* = 18), and eight quality maternity care practices (*n* = 641) were carried out using three separate tools. Willing maternity staff (*n* = 237) also completed a survey on their knowledge, perceptions, and use of the maternity care interventions. Descriptive statistics and logistic regression were used to identify differences between the hospital types. The qualitative component comprised six focus groups and 18 interviews involving midwives, other maternity staff, and managers from the three hospital types. Data were analysed using an inductive cyclical process of immersion and iteration to draw out themes. The quantitative and qualitative methods complemented each other and were used synergistically to identify the study’s insights.

**Results:**

Quantitative analysis found that, of the eight quality practices, hospitals with midwives but no mentors were significantly more likely than hospitals without midwives to use three: upright labour (94% vs. 63%; OR = 22.57, *p* = 0.001), delayed cord clamping (88% vs. 11%; OR = 140.67, *p* < 0.001), skin-to-skin (94% vs. 13%; OR = 91.21, *p* < 0.001). Hospitals with mentors were significantly more likely to use five: ANC card (84% vs. 52%; OR = 3.29, *p* = 0.002), partograph (97% vs. 14%; OR = 309.42, *p* = 0.002), upright positioning for labour (95% vs. 63%; OR = 1850, *p* < 0.001), delayed cord clamping (98% vs. 11%; OR = 3400, *p* = 0.003), and skin-to-skin contact following birth (93% vs. 13%; OR = 70.89, *p* < 0.001) Qualitative analysis identified overall acceptance of midwives and the transition to improved quality care; this was stronger with facility mentoring. The most resistance to quality care was expressed in facilities without midwives. In facilities with midwives and mentoring, midwives felt proud, and maternity staff conveyed the greatest acceptance of midwives.

**Conclusion:**

Facilities with professional midwives had better availability and quality of maternity care across multiple components of the health system. Care quality further improved with facility mentors who created enabling environments, and facilitated supportive relationships between existing maternity staff and managers and the newly deployed midwives.

**Supplementary Information:**

The online version contains supplementary material available at 10.1186/s12884-022-05096-x.

## Background

Despite decades of global prioritization, pregnancy-related morbidity and mortality remain a significant public health and human rights concern for the world’s poorest [1]. Between 2000 and 2017, the global maternal mortality ratio (MMR) fell by 38%, from 342 to 211 deaths per 100,000 live births. Southern Asia experienced the largest regional drop in MMR, with a reduction of 59% from 384 to 157 deaths per 100,000 live births [2]. Bangladesh has notably reduced its MMR from over 500 deaths per 100,000 live births in 1980, to the current rate of just under 200 per 100,000 live births. However, since 2010 MMR has stagnated [3].

A critical challenge is that, as MMR declines, further reductions become more difficult to achieve. Care quality and availability, health systems challenges, and socioeconomic determinants of health make up a multi-layered context where significant change is needed to continue to advance progress [4]. Professional midwives are an essential cadre to invest in to address these challenges. They offer the advantage of being lower cost, involving fewer medical interventions, and leading to more positive childbirth experiences for women with equal or improved health outcomes [5]. It is estimated that a substantial increase in coverage of midwives educated to international standards and working in an enabling environment could avert 41% of maternal deaths, 39% of neonatal deaths, and 26% of stillbirths [6].

Yet, significant gaps exist in midwives educated to global standards, and working in enabling environments, in low- and middle-income countries (LMICs). The potential of midwives to improve quality of care in these settings is yet to be fully realized [7]. There are knowledge gaps on both the impact of professional midwives in LMIC health systems, and program approaches that successfully address enabling environments. This is in part because the majority of existing research on midwifery interventions in LMICs does not use a standard definition for a midwife [8, 9].

The International Confederation of Midwives (ICM) defines a professional midwife based on standard pre-service education and a scope of practice that includes a focus on women’s right to quality maternal health care [7]. While forecasts on the potential impact of midwives are based on this standard, actual learnings on ICM-standard midwife programs in LMICs have not yet been thoroughly documented [10]. This paper uses the term midwife to describe diploma prepared midwives educated to ICM standards.

This study examined if the introduction of professional midwives was associated with improved availability and quality of maternity care provision in 12 sub-district government hospitals in Bangladesh. It also documented the experiences of the midwives, as well as the maternity staff and managers they joined, in navigating barriers and facilitators to midwives serving as autonomous maternal health care providers. The aims of this research were 1) to determine if introducing international standard midwives in rural sub-district hospitals in Bangladesh, both with and without mentoring, was associated with improved availability and quality of maternal and newborn health care; and 2) to explore the experiences of the midwives, and the other maternity staff and managers, following their introduction. Key objectives were to examine the enabling environment and document barriers and facilitators to midwives providing quality care. This research sought to document lessons from implementation to inform similar work in other countries, and expand the global body of knowledge on introducing globally standard midwives distinct from nurses in LMICs.

## Methods

This study employed a mixed-methods observational design to examine differences in care practices and maternity staff experiences and attitudes between three distinct categories of government sub-district hospitals: those without midwives, those with midwives, and those with midwives and facility mentors. The care practices recommended by the World Health Organization (WHO) in *Standards for improving quality of maternal and newborn care in facilities* and *WHO recommendations on antenatal care for a positive pregnancy experience* were used to frame the analysis [11, 12]. In accordance with these guidelines, evidence-based routine birth care includes 1) respectful and woman centred care; 2) no routine use of oxytocin, episiotomy, lithotomy position, or caesarean section; and 3) routine use of skin-to-skin contact, delayed umbilical cord clamping, companionship, partograph, active management of the third stage of labour, upright position for labour and birth, and oral hydration and nutrition in labour.

Two types of quantitative data collection approaches—survey and observation—and two types of qualitative approaches—interview and focus group discussions—were used for triangulation. Quantitative and qualitative data were collected concurrently with equal weight placed. The lead researcher, a female Certified Nurse Midwife and PhD, lived and worked in Bangladesh, in part with public hospital maternity services. Therefore, enough was known to develop the quantitative and qualitative components and use them synergistically to deepen understandings, rather than use one to inform the design of the other. All four datasets were compared and contrasted to find relationships and associations within and between the different groups. Important insights were gleaned from both the quantitative and the qualitative data individually, as well as from the analytic conversations between them. The results were not weighted toward either method, but rather analysed equally to draw out a range of insights.

Data collection tools were developed by the lead researcher based on existing evidence-based surveys. All data collection tools are included with supplementary material. Recently graduated midwives, junior to those working in the government facilities, were hired by the lead researcher as research assistants for data collection, translation and transcription. The lead researcher provided them with training and supervision. The professional role of the lead researcher created the potential for researcher bias. Likewise, the participants may have felt the need to modify what they said in order to answer questions according to what they perceived the researcher may have wanted to hear. While only some of the participants in the study were aware of the lead researcher’s role as someone who supports the midwifery profession and mentoring projects through her work with the United Nations (UN), others potentially became aware during the research [13]. Reflexivity, or self-awareness of intentions and process, was used to mitigate these potential biases through adhering to transparent field notes, defined methods of analysis, and open discussion. The research was overseen by two PhD prepared faculty from Lancaster University in the UK. Approval for the study was obtained from national government authorities at the Directorate General of Health Services. Following preparation of data collectors, all tools were piloted in a government sub-district hospital and then modified slightly for clarity of information gathered [14]. Data collection occurred in April and May of 2019. Research announcements were posted at each hospital two weeks prior to the researchers’ visit. On the day of data collection, the research team met with all hospital staff and managers to review the study purpose and data collection process. Field notes were completed at the end of each day.

### Study setting

There are gaps in maternity care quality and availability in Bangladesh at all levels of the health system. To respond to the stagnating MMR, in 2013 the Government of Bangladesh commenced a standard ICM-aligned diploma in midwifery program in 20 nursing colleges. This is notable as it is a country-wide initiative led by the government, as opposed to a sub-national or agency-led project. This research thus observed pre-existing government interventions as a natural experiment. Before midwives’ introduction, nurses, in collaboration with doctors, were providing maternity care. Nursing education programs in Bangladesh include content on midwifery, but not to the level of meeting ICM standards for midwifery. In August of 2018, nine months prior to this study, the first class of midwives deployed to rural sub-district hospitals [15] known to care for the poorest who bear the highest burden of maternal mortality [16]. Sub-district hospitals in Bangladesh are standardized in that the building infrastructure, allocated medicine and equipment, and staffing plans are identical. Midwife deployment was staggered such that some hospitals began employing midwives prior to others. To support the midwives to transition into their new roles, a project mentorship programme funded by UNFPA was introduced in selected health facilities. This was necessary, as newly deployed midwives were young new graduates and it was anticipated that they would need support to transition into their new roles and bring about quality changes. Mentors were selected from among female medical graduates to operate as peers of the managers in order to create enabling environments for midwifery within hospitals. Mentors received a 1-week orientation on the role of midwives, and the latest WHO quality maternity care guidelines with semi-annual update training and ongoing access to midwifery experts. The programme consisted of bi-monthly visits in which mentors met with managers and staff to guide and support appropriate use of midwives and improved clinical care implementation [17, 18].

### Hospital selection

In the initial 2018 deployment, 1,149 midwives deployed to 342 of the country’s 430 sub-district hospitals. Four midwives were planned for each facility. The mentoring project was initiated in 50 of the hospitals. Subsequent deployment took place after the data were gathered for this study. To meet the criteria for inclusion in this study as a hospital without midwives, it had to have no midwives deployed. To meet the criteria of having midwives, four midwives needed to be providing care. The country's 19 busiest sub-district hospitals from each hospital group were identified and recruited to participate in the study. Service delivery numbers were determined through using the government district health information system (DHIS2) records and then contacting the hospitals to confirm the information. Fourteen of the 64 districts, and seven of the eight divisions were represented. Although most of the districts were in the country’s predominate flat river delta area, chosen districts were also from coastal, hilly, and flood plain areas that tend to be harder to reach [15, 18].

### Quantitative

#### Design

The quantitative component of the study utilized observations of facility readiness and implementation of selected birth practices within the three categories of hospitals. The birth practices were upright positioning for labour and birth, companionship and hydration during labour, avoidance of episiotomy and manual exploration of the uterus, delayed cord clamping, and skin-to-skin contact between mother and baby for one hour following birth. A survey of maternity care providers’ and managers’ perceived knowledge, attitudes, and reported use of clinical behaviours as related to quality maternity care was also done.

#### Sample

Convenience sampling was used for the quantitative component of this study as both staffing and patient flow remain consistent and homogeneous throughout the week. For facility readiness, the selected hospitals made up the sample. For the clinical observations and survey, participants were chosen based on their availability. Non-participation was not tracked, however, numbers of participants roughly matched what would be expected if all those eligible participated [19]. As the study was conducted on working nurses and doctors, the primary reason for non-participation was being busy with patient care.

- For clinical observations, the sample size was determined through power analysis to find the minimum number of observations needed to detect significant differences in implementation of the observed WHO quality care interventions between three groups. Using an alpha of 0.05 and beta of 0.8, a total sample size of 159 observations was recommended in order to detect a medium size effect (f = 0.25). The sample included all consenting maternity staff who were conducting antenatal care and or births, as well as all pregnant and immediate postpartum mothers receiving care during the observations. A total of 169 women agreed to participate in the study’s labour room observations: 54 in the no midwives group, 51 in the midwives without mentoring group, and 64 in the midwives and mentoring group. Additionally, 473 women attending antenatal care (ANC) consented to observation. Each sample exceeded the 159 required to detect a medium effect size as determined in the power analysis.
- All available maternity staff and managers were invited to take the survey; 237 maternity staff and managers consented and completed the survey.

#### Tools

The three quantitative data tools examining evidence-based maternity care practices were: 1) a hospital readiness tool, 2) a clinical observation tool, and 3) a survey. The facility readiness tool consisted of direct observations of medicines and supplies, and service utilization data logged in register books. Binary observational data were gathered in the clinical observation tool, which included instructions to guide researchers to identify and record the behaviours being measured. Measurement was “yes” or “no”. “Yes” denoted use of selected evidence-based care interventions or presence of an aspect of facility readiness. Contrarily, “no” denoted lack of use/presence. Observations were made at unannounced times to reduce the risk of the Hawthorne effect [20].

The survey was developed based on an existing evidence-based practice survey tool. The original tool was designed for nurses and had been validated, though it was not specific to maternity care [21]. For this study, the content was adapted for maternity care quality using the WHO guidelines. Questions explored perceived knowledge, capacity, use, and value, of evidence-based maternal and newborn healthcare interventions. Some question formats used were identical to the original tool and others were slightly modified. The survey was written in English and Bengali. Translation was conducted from English to Bangla and then Bangla to English by two professional translators. Survey responses were largely provided in English but when needed translation was provided by the researchers.

#### Data collection

The research assistants carried the observation tools and waited in the antenatal and birth care areas of the hospitals to recruit participants. They asked all hospital staff and managers to complete surveys and performed observations based on availability of women receiving antenatal care and giving birth. The research assistant midwives remained onsite for up to 10 days after the larger research team had departed in order to observe a minimum of 10 births. During the observations, researchers had access to the birthing woman throughout labour, birth and up to 2 h post-partum. They did not provide labour support to the birthing woman or guidance to the maternity staff. Apart from obtaining consent and entering and exiting when needed to take breaks, interactions with participants were limited. As the various birth interventions (i.e., the variables of interest) were carried out by birth attendants, the researchers would check them off on the observation tool. Specific instructions were given to researchers for what needed to be observed in order to count a practice as having been done. For example, to check off that labour was non-supine, a birthing woman had to labour 90% of the time in an upright or lateral position. Delayed cord clamping meant that the umbilical cord was cut only after it stopped pulsing. To check off skin-to-skin, it must have been practiced between mother and baby for one hour. All staff and patients were informed of the general research aims and objectives, though the names of the specific birth practices being observed were not discussed.

#### Data analysis

Data from the facility readiness tool and the survey were analysed using descriptive statistics to identify patterns and trends across hospital types. Missing data from the observations of specific birth practices were excluded from the analysis of the associated outcomes. The clinical observation data were analysed using logistic regression. Fixed-effect and mixed-effect logistic regression models were implemented using the *lme4* package in R statistical language [22, 23] to determine whether there were significant differences in application of the WHO guided maternity practices.

In the logistic regression, frequency of use of upright positioning for labour and birth, companionship and hydration during labour, avoidance of episiotomy and manual exploration of the uterus, delayed cord clamping, and skin-to-skin contact between mother and baby were the outcomes of interest measured from the clinical observations. The main predictors of interest were the presence of midwives and mentoring. Results are presented unadjusted and also adjusted for hospital level co-variates. The co-variates included were the average experience of providers measured from the survey and number of deliveries from the hospital readiness form as these could have affected the clinical care provided.

### Qualitative

#### Design

Focus groups and interviews were held with midwives, other maternity staff, and hospital managers. The focus groups and interviews with the maternity staff and managers sought input with regard to their experiences related to the new midwives and the improved quality of care the midwives brought. The focus groups with midwives gathered views on their new roles and their success and challenges in implementing quality improvements. Across participants, they illuminated the experience of the different groups of health care providers; they allowed for the development of understanding on maternity care providers’ ideas, how they interacted on the different topics, and their collective understanding. Interviews were largely used for the busy hospital managers and doctors who were less likely to be willing to participate in focus groups. The topics for the focus groups and interviews were similar, but managers were more open when they could share their perceptions and feelings alone.

With attention to reflexivity, the researcher attempted to be transparent at every step and aware of the possibility influencing the conversations with participants, and in the analysis of the data [13]. Because this research discusses improving the quality of care, and conversations with maternity staff and managers who may not have been providing optimum quality, care was given to protecting the vulnerability of participants. At the same time, efforts were made to illicit genuine, substantive interactions about motivating drivers and what works for change [13]. Rigor was strengthened through triangulation between the four methods of data collection. In addition, as over 50 people were involved in focus groups and interviews, many voices were heard, providing an opportunity for a variety of perspectives.

#### Sample

The sampling for the focus groups and interviews was purposeful. Purposeful sampling is a method of selecting participants based on the clients’ past experiences or knowledge and allows researchers to choose information rich cases [24]. Participants were primarily middle-class Bangladeshi Muslims health care workers who lived in the community served by the hospital, and were educated as generalist doctors, obstetricians, nurses, and midwives. A sample size of 6–8 participants per focus group was chosen for feasibility. Six focus group discussions allowed for both midwives and maternity staff from each facility type to be interviewed. Each hospital type had one focus group for midwives (where relevant), and one for other maternity staff who consisted of nurses and doctors. One additional focus group was conducted at hospitals with midwives and mentors, and a decision was made to use the content as data saturation was not considered to have been reached. There was a total of ± 40 participants in focus groups; all were female. Eighteen individual interviews were conducted with the managers as they were time constrained and, given the cultural hierarchy, more likely to participate in a one-on-one discussion. There were three types of available managers—a hospital manager (all male), a nursing and midwifery manager (all female), and a head obstetrician (some female; some male). Two of each manager type from each hospital type were interviewed to have some comparison and variety. No eligible participants who were approached to participate in the study declined, nor did any drop out.

#### Tools

Focus group discussions and interviews followed a semi-structured interview guide developed by the lead researcher. Questions for non-midwife staff and managers explored if they had made any quality improvements recently, what they were, and what facilitated them. If the facility had midwives deployed, respondents were also asked how they felt about the midwives. Questions in midwife facilities sought whether evidence-based care interventions were used and how providers felt about those interventions, management of obstetric emergencies, if the midwives had made changes (and if so, what changes), the scope of the midwives’ practice and how they felt about it. They also explored how they felt about mentorship, what changed with mentorship, and what changed with the introduction of the midwives. In addition to the relevant questions above, the midwives were asked how they felt in their new roles implementing various aspects of quality care and introducing new clinical interventions, including challenges.

#### Data collection

Focus group discussions and interviews were conducted with maternity staff and managers during work hours, as holding them after office hours did not seem to be an option–many staff lived far away and valued time off. As a result, discussions were short in length with focus groups averaging 36 min (standard deviation 5 min) and interviews averaging 20 min (standard deviation 10 min). The lead researcher conducted the focus groups and interviews. Privacy was maintained as all discussions were held in a room with a closed door. The interviews and focus groups were all facilitated in English with Bangla translation provided by translators. The researcher posed questions back to participants following their comments when clarification was needed, and the information shared during the discussion was paraphrased at appropriate times during the discussions to allow participants the opportunity to validate what was said or correct the researcher’s understanding. English transcriptions were developed by the translator based on recordings of the conversations. These transcriptions were shared with the contributing participants who expressed interest given the language barrier. Although the translators' ability to translate concepts appeared to be satisfactory, English grammar and spelling were imperfect. To address this for ease of reading, corrections to some of the quotations were made by the researcher.

#### Data analysis

Information addressing the research questions was analysed inductively. The intention when analysing the data was to be curious about what new information was arising as opposed to looking for patterns that fit into existing theories. Transcriptions were studied using context analysis, a method of listening for a sense of the whole rather than fracturing data into pieces [25, 26]. The qualitative data were analysed following Bazeley 2013 [27], in which an iterative process of data reduction and display through reading, reflecting, and seeking out emergent themes was used to capture a sense of the whole picture. The software programme NVivo was used. The lead researcher carried out the coding, which consisted of reading the transcripts and identifying topics or words that participants repeated. The most representative quotations that covered both the breadth of the ideas expressed, and that represented the general proportion of that sentiment within the themes from each of the facility levels and staff and managers were chosen. Eighty-six codes were identified and sorted into separate folders in NVivo. Themes were separated by hospital type, and into midwives as opposed to other maternity staff, to compare and contrast the shared experiences. There were thus five different potential categories for each theme (Table 1).

**Table 1:** Example of the coding process for the theme, “resistance to change”

Theme	Potential categories
Resistance to change	No midwives - Only non-midwives Midwives - Experiences of the midwives - Experiences of the non-midwife maternity staff and managers Midwives with mentoring - Experiences of the midwives - Experiences of the non-midwife maternity staff and managers

The coded data were then combined into sub-themes and grouped into 10 overall themes organized around: 1) maternity staff's and managers’ perceptions and experiences related to the new midwives’ service provision, and 2) the midwives’ own experiences of moving into their new roles. An example of the quotations, codes and themes for the ‘resistance to change’ theme is provided in the supplementary material (Table S1). The 10 initial themes were later slightly modified for clarity.

## Results

### Quantitative

The quantitative results comprised 19 hospital readiness observations, 641 clinical observations, and 237 completed surveys. Table 2 shows the breakdown of hospitals by division, numbers of births in the six months prior to the study, and numbers of observations of ANC sessions and births. In hospitals without midwives, with midwives only, and with midwives and mentorship, 2,343, 2,527, and 5,559 births took place, respectively. Monthly births in the sampled hospitals remained relatively stable in the six months prior to data collection. Observations of ANC and births were of nurses and midwives providing care. Survey respondents by profession are shown in Table 3.

**Table 2:** Hospital births and observation numbers

	#	Division	Births Oct '18-Mar '19	Observations	
				ANC	Births
**No midwives**	1	Sylhet	222	21	11
	2	Khulna	290	25	9
	3	Dhaka	188	22	10
	4	Rangpur	377	2	12
	5	Rangpur	760	36	10
	6	Sylhet	331	19	10
	7	Sylhet	175	2	2
		**Total**	**2,343**	**127**	**64**
**Midwives**	8	Chittagong	509	25	11
	9	Rangpur	603	47	10
	10	Khulna	504	28	10
	11	Chittagong	449	20	10
	12	Mymensingh	462	21	10
		**Total**	**2,527**	**141**	**51**
**Midwives + mentors**	13	Khulna	886	28	10
	14	Chittagong	1,185	20	12
	15	Moulvibazar	769	29	13
	16	Rangpur	319	76	10
	17	Rajshahi	776	30	9
	18	Mymensingh	1,624	22	10
		**Total**	**5,559**	**205**	**64**

**Table 3:** Survey respondents by profession

	**Provider type**		
**Hospital type**	Nurses	Midwives	Doctors
No midwives	78	0	18
Midwives	40	16	4
Midwives + mentors	45	28	10
**Total**	**163**	**44**	**32**

#### Hospital readiness: equipment, supplies, and separated antenatal care (ANC) service provision

**Fig. 1:**
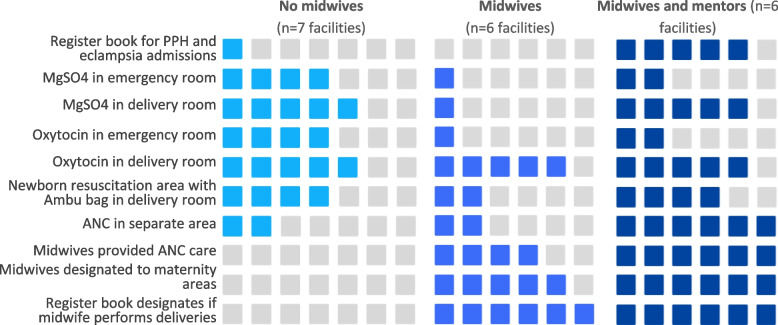
Availability of emergency equipment and supplies, and separate ANC service provision

Readiness checklists completed at each hospital revealed differences between the hospital types in availability of equipment and supplies for responding to obstetric emergencies, as well as in whether ANC service areas were distinct from general female consultation areas, ANC cards were used, and whether midwives—as opposed to doctors or nurses—provided the service (Fig. 1).

As shown in Fig. 1, overall, facilities with midwives and mentorship were better prepared than the other two hospital types. Of the seven hospitals without midwives, four had the infrastructure and supplies to be able to respond to obstetric emergencies as they had oxytocin and magnesium sulphate in either the emergency or birthing rooms and they also had a newborn resuscitation area with an Ambu bag in the birthing room. One of the six hospitals with midwives met this criteria, as did five of the six with both midwives and mentors. Large differences between hospitals with midwives and those with midwives and mentors were observed for having a midwifery-led ANC clinic distinct from general consultations.

While oxytocin was widely available in birthing rooms in all hospital types, this was likely due to its routine use in the post-partum period as well as in labour augmentation. In mentored hospitals, oxytocin and magnesium sulphate were present in five out of six hospital birthing rooms, but in only two out of six emergency rooms. This may be indicative of a practice difference occurring specifically in mentored hospitals in which management of obstetric emergencies was transferred from the emergency room to the birthing rooms and then treated by midwives.

#### Observations of care

**Fig. 2:**
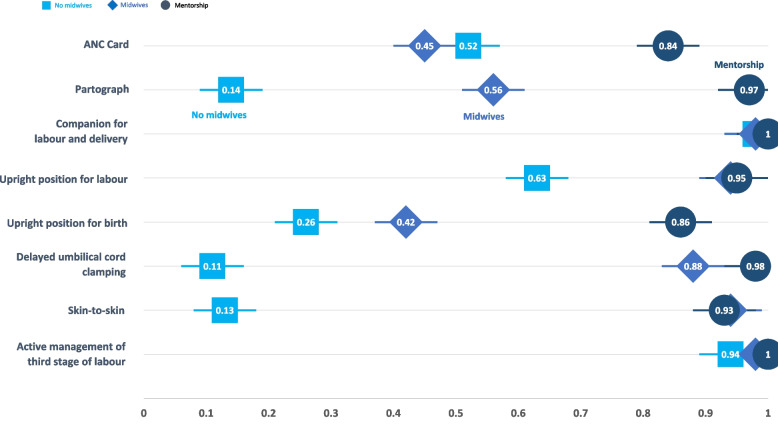
Clinical observations by hospital type

Across the three types of hospital settings, a continuum was identified with less use of evidence-based practises in hospitals without midwives and increasingly more use across hospitals with midwives and hospitals with midwives and mentors. Figure 2 provides a visual depiction of the practice differences in the three hospital types using clinical observation data.

The logistic regression models examined the clinical observations of evidence-based care practices across the hospital categories to determine whether there were indications of significant differences. Results from the fixed-effect model adjusted for hospital level co-variates are shown in Table 4 —asterisks next to the model results indicate statistically significant differences in relation to the reference group (i.e., hospitals without midwives). The model without co-variates is shared in supplementary material. Considering that eight tests were conducted, a Bonferroni adjusted alpha of 0.00625 was applied to correct for cumulative error when multiple tests are conducted on one sample [28].

**Table 4:** Odds ratios and 95% confidence intervals for fixed-effect logistic regression models (with co-variates)

	*Dependent variable*							
	**ANC Card**	**Partograph is used**	**Upright lateral labour**	**Companion present**	**Delayed cord clamping**	**Skin-to-skin contact (1hr)**	**Active management of the third stage of labour**	**Upright lateral birth**
**Intercept **	4.31^**^ (1.98, 9.39)	14.96^*^ (1.61, 139.34)	21.76^*^ (1.7, 278.69)	142.25 (0.02, 1.18e+06)	0.05 (0e+00, 1.44)	0.2 (0.02, 1.97)	78.47 (0.15, 4.13e+04)	1.21 (0.23, 6.39)
**Midwives without mentors**^**a**^	0.56^*^ (0.32, 0.97)	4.84^*^ (1.28, 18.32)	22.57^**^ (4.07, 125.07)	1.01 (0.02, 51.13)	140.67^**^ (20.11, 983.94)	91.21^**^ (17.73, 469.19)	1.87 (0.09, 38.59)	1.19 (0.43, 3.27)
**Midwives with mentors**^**a**^	3.29^**^ (1.61, 6.74)	309.42^**^ (8.77, 1.09e+04)	1.85e+03^**^ (32.56, 1.05e+05)	1e+08 (0e+00,$\infty$)	3.4e+03^**^ (52.41, 2.2e+05)	70.89^**^ (7.96, 631.31)	3.1e+07 (0.00-$\infty$)	6^*^ (1.33, 27)
**Hospital Avg. Experience (Years)**	0.8^**^ (0.71, 0.9)	0.49^**^ (0.34, 0.73)	0.91 (0.65, 1.29)	0.94 (0.29, 3)	1.26 (0.81, 1.96)	0.95 (0.68, 1.34)	0.81 (0.38, 1.73)	0.78 (0.61, 1.01)
**Hospital Deliveries**	1.01 (1, 1.01)	1.02 (0.99, 1.04)	0.97^*^ (0.95, 0.99)	0.99 (0.93, 1.06)	0.98 (0.96, 1.01)	1 (0.98, 1.02)	1.01 (0.97, 1.05)	1.01 (1, 1.03)
**Number of observations**	472	166	168	169	159	161	164	160

⋆*p*<0.05; ⋆⋆*p*<0.00625 (Bonferrroni-adjusted alpha)

^**a**^Reference category: no midwives

Missing values are reflected in Table 4 in the variability in numbers of observations. As up to eight practice observations were to be carried out for each birthing woman, research assistants missed some observations due to engagement in personal activities (e.g., eating, sleeping or using the restroom). There were more missing observations for activities occurring during and immediately following birth—delayed cord clamping, upright lateral birth and skin-to-skin—due to the relatively short period of time within which these can be observed.

The fixed-effect analysis showed that, compared to hospitals without midwives, hospitals with midwives and mentors were significantly more likely to use five of the eight new evidence-based practices: ANC card (84% vs. 52%; OR = 3.29, *p* = 0.002), partograph (97% vs. 14%; OR = 309.42, *p* = 0.002), upright positioning for labour (95% vs. 63%; OR = 1850, *p* < 0.001), delayed cord clamping (98% vs. 11%; OR = 3400, *p* = 0.003), and skin-to-skin contact following birth (93% vs. 13%; OR = 70.89, *p* < 0.001). The degree of effect varied with ANC card use being 3.29 times more likely and delayed cord clamping being 3400 times more likely. Hospitals with only midwives were significantly more likely to use three of the eight: upright labour (94% vs. 63%; OR = 22.57, *p* < 0.001), delayed cord clamping (88% vs. 11%; OR = 140.67, *p* < 0.001), and skin-to-skin (94% vs. 13%; OR = 91.21, *p* < 0.001). Overall, odds ratios for variables from hospitals with mentorship were larger than those from hospitals with only midwives, indicating a greater likelihood of these practices being used when mentors were present. A mixed-effect regression model was also employed in order to control for unknown factors within hospitals that may have had an influence on outcomes (available in supplementary material). Though the number of hospitals in the sample size was about 25% of the size needed for a mixed-effect model to have adequate power, five practices were still significantly more likely to be used in hospitals with mentorship compared to those without midwives. These were: ANC card, partograph, upright labour, delayed cord clamping, and skin-to-skin contact. Three practices were significantly more likely to be used in hospitals with midwives without mentorship compared to those with no midwives: upright labour, delayed cord clamping and skin-to-skin contact. Applying the Bonferroni adjustment to the mixed-effect results reduced the number of significantly more likely practices to two in hospitals with mentorship (upright labour and delayed cord clamping) and one in hospitals with only midwives (delayed cord clamping).

#### Survey

Overall, maternity staff's and managers’ self-reported survey responses on their valuing and perceptions of evidence-based care practices revealed less acceptance in hospitals without midwives than maternity staff and managers in the other facility types. Summary results from survey questions on maternity staff's and manager's value of evidence-based practices were largely homogeneous, though with some interesting variation. For example, almost all participants agreed or strongly agreed that partographs were helpful, that companionship during labour was important, and that skin-to-skin after birth was the best care for babies. However, there were notable differences in terms of delayed cord clamping and non-supine positions. Survey results are presented in Tables 5 and 6.

**Table 5:**
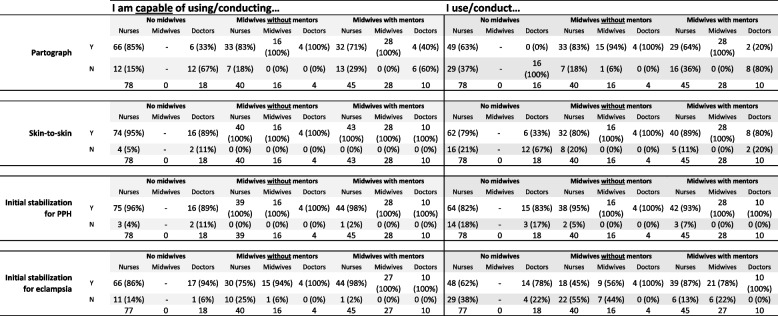
Detailed capabilities and actions, by hospital and provider type

**Table 6:** Detail on provider agreement on use of evidence-based practices

**Partograph is helpful**	**Strongly agree**	**Agree**	**Neutral**	**Disagree**	**Strongly disagree**
No midwives	92 (89%)	9 (9%)	2 (2%)	0 (0%)	0 (0%)
Midwives without mentorship	64 (93%)	5 (7%)	0 (0%)	0 (0%)	0 (0%)
Midwives with mentorship	80 (89%)	9 (10%)	0 (0%)	0 (0%)	1 (1%)
**A companion during labor and delivery is a good idea**	**Strongly agree**	**Agree**	**Neutral**	**Disagree**	**Strongly disagree**
No midwives	87 (84%)	14 (13%)	0 (0%)	2 (2%)	1 (1%)
Midwives without mentorship	64 (91%)	6 (9%)	0 (0%)	0 (0%)	0 (0%)
Midwives with mentorship	75 (84%)	12 (13%)	1 (1%)	0 (0%)	1 (1%)
**Delayed cord clamping is a good idea**	**Strongly agree**	**Agree**	**Neutral**	**Disagree**	**Strongly disagree**
No midwives	35 (34%)	32 (31%)	3 (3%)	6 (6%)	28 (27%)
Midwives without mentorship	30 (43%)	15 (21%)	5 (7%)	4 (6%)	16 (23%)
Midwives with mentorship	69 (78%)	11 (12%)	1 (1%)	5 (6%)	3 (3%)
**Non supine position is important for pregnant and labouring women**	**Strongly agree**	**Agree**	**Neutral**	**Disagree**	**Strongly disagree**
No midwives	29 (29%)	39 (39%)	2 (2%)	7 (7%)	24 (24%)
Midwives without mentorship	29 (42%)	19 (28%)	6 (9%)	4 (6%)	11 (16%)
Midwives with mentorship	66 (75%)	14 (16%)	3 (3%)	4 (5%)	1 (1%)
**Skin-to-skin contact for one hour after delivery is the best care for mother and baby**	**Strongly agree**	**Agree**	**Neutral**	**Disagree**	**Strongly disagree**
No midwives	85 (82%)	16 (15%)	0 (0%)	0 (0%)	3 (3%)
Midwives without mentorship	65 (93%)	5 (7%)	0 (0%)	0 (0%)	0 (0%)
Midwives with mentorship	77 (86%)	11 (12%)	0 (0%)	1 (1%)	1 (1%)
**Having Diploma midwives in the ANC and maternity area is the best care for mother and baby**	**Strongly agree**	**Agree**	**Neutral**	**Disagree**	**Strongly disagree**
No midwives	58 (74%)	15 (19%)	2 (3%)	2 (3%)	1 (1%)
Midwives without mentorship	63 (90%)	7 (10%)	0 (0%)	0 (0%)	0 (0%)
Midwives with mentorship	78 (52%)	71 (47%)	1 (1%)	0 (0%)	0 (0%)
**If your facility participated in the Save the children (SCI) mentorship, was it helpful**	**Strongly agree**	**Agree**	**Neutral**	**Disagree**	**Strongly disagree**
No midwives	5 (12%)	6 (14%)	1 (2%)	1 (2%)	29 (69%)
Midwives without mentorship	23 (68%)	1 (3%)	4 (12%)	2 (6%)	4 (12%)
Midwives with mentorship	69 (80%)	16 (19%)	1 (1%)	0 (0%)	0 (0%)
**Recent introduction of Diploma midwives is helpful**	**Strongly agree**	**Agree**	**Neutral**	**Disagree**	**Strongly disagree**
No midwives	12 (26%)	6 (13%)	1 (2%)	0 (0%)	28 (60%)
Midwives without mentorship	56 (80%)	14 (20%)	0 (0%)	0 (0%)	0 (0%)
Midwives with mentorship	75 (99%)	1 (1%)	0 (0%)	0 (0%)	0 (0%)

### Qualitative

Eighteen interviews and six focus group discussions were conducted with midwives, and other maternity and emergency staff caring for pregnant women. The interviews and focus groups revealed some similarity among respondents across the three hospital types in terms of feelings regarding midwives, and experiences related to transitioning to more evidence-based care. However, disparity between the groups was more commonly identified. The disparity largely corroborated the already identified continuum of greater appreciation of evidence-based practices in hospitals with midwives, which improved with the presence of mentors. The themes that emerged from the qualitative analysis are summarized in Table 7 . Within most of the themes, 10–15% of participant comments expressed discordant views.

**Table 7:** Themes that emerged from the qualitative data

**Theme**	**Description**
**Imagined and experienced benefits of midwives**	Awareness among nurses and managers that midwives could be helpful was notable in that, where there were no midwives, the imagined benefits were overwhelmingly positive, whereas, where there were midwives but no mentoring, most saw the midwives as too inexperienced and not capable enough to make positive change. This is a significant finding as it leads to midwives’ scope of practice being limited by their supervisors. Where there was facility mentoring, those that commented on this topic saw the midwives as beneficial.
**Familiarity with and use of improved care quality**	Midwives and mentors were associated with increased comfort with, and use of evidence-based care. This theme plays out across the continuum in that, where there were no midwives and where there were midwives and no mentors, nurses had some familiarity with WHO standard quality maternity care, but they were not comfortable using it. When midwives were introduced, all midwives expressed comfort, but some were not using it. With mentoring, the nurses were more comfortable, and the midwives were enabled to use the quality-of-care practices; thus, all stated they were providing quality care.
**Resistance to change**	Entrenched habits, social/patient/family pressure, and under-the-table payments were found to lead to resistance to change. This theme also found a continuum where non-midwife maternity staff and managers in hospitals without mentors expressed similar levels of resistance to change, but with mentoring there was much less resistance. Most midwives wanted change, but without mentoring many were complacent with existing systems. With mentoring most midwives felt they were making change.
**Under-the-table fees**	Under-the-table fees were a cause for increased competition between nurses and midwives as nurses lost tips if they turned over the maternity area to the midwives. In addition, the desire to provide free care for the poor arose spontaneously from some of the midwives. Mangers identified charging fees as a limitation for caring for the poor and as the reason why nurses did not want the midwives to move into autonomous roles.
**Management of obstetric emergencies**	Non-midwife maternity staff described numerous barriers to caring for women with obstetric emergencies. Midwives talked about being competent and willing to manage obstetric emergencies, but those without mentoring often spoke of resistance from managers. With mentoring, most stated that they were managing emergencies.
**Barriers and facilitators to midwives practicing autonomously and to their full scope**	A number of issues were identified as barriers to midwives practicing autonomously. Most commonly, youth and/or inexperience were mentioned. Managers mentioned competition between nurses and midwives limiting the midwives. Midwives spoke of not having their own separate units. Mentoring was seen by many as facilitating relationships between nurses and midwives.
**Maternity staff's, managers’, and midwives’ perceptions of midwives' competence to move into their role**	Perceptions of midwives' lack of competence were expressed as a reason to limit midwives' autonomy. This was particularly notable where there was no mentoring. Nurses and midwives expressed that women were concerned about midwives’ competence. This was less prevalent in hospitals with mentoring. Midwives consistently perceived themselves as competent.
**Midwives' pride**	Midwives spontaneously expressed that they felt pride in providing good care to the poor. This was true in both mentored and non-mentored sites.
**The experience of mentorship by hospital staff**	The hospital staff reported a greater sense of having a supportive team, and a better understanding of midwives' competencies with mentorship.
**Midwives and other maternity staff and managers desire to care for the poor**	Midwives spontaneously expressed that they wanted the poor to know that they would care for them for free. No other maternity staff or managers expressed this, though some managers and non-midwife maternity staff spoke of the limitations regarding caring for the poor.

#### Hospitals without midwives

Both nurses and managers in hospitals without midwives expressed the view that midwives would be able to fill a quality gap. They viewed midwives, over the existing nurses, as specially trained maternity care providers who would be able to improve outcomes. They also expressed a lack of knowledge and/or use of evidence-based maternity care. For example, while verbal recognition was given to the importance of quality ANC, at most of the hospitals staff described not having ANC services separate from other care. While some respondents spoke about the benefits of family companionship during labour and birth, others expressed concern about companions being difficult or demanding. All described that women deliver exclusively in supine positions. All expressed support for skin-to-skin between mother and baby, though most also shared that it increases their workload. Under-the-table tipping was also discussed. One hospital manager described that, while he feels motivated to serve the poor, most cannot afford the under-the-table fees that are commonly required.

As one nurse shared,

*“We perform skin-to-skin only for one minute because one hour is not comfortable, it is not possible to conduct skin-to-skin contact for an hour … We do other work and there is a lack of manpower.”*

- Nurse 3, no midwives

In all hospitals without midwives, respondents described avoiding stabilization for obstetric emergencies. Although some nurses said that they provide initial treatment, most stated that if the situation is critical, they only refer women to another (higher-level) hospital. The reasons behind maternity staff referring without stabilizing or providing initial treatment include feeling inadequately equipped with the resources to provide the needed care and as a result being concerned about women dying (and thus angering the community), and appearing in an unfavourable light in reports to authorities.

#### Hospitals with midwives

Across hospitals with midwives only, maternity staff made somewhat more positive statements about WHO guided quality care interventions. Doctors and nurses spoke of needing more training, being comfortable with existing habits, and having too much work pressure and not enough time to implement the needed changes. They also spoke of women’s families not wanting the evidence-based care practices. For example, statements regarding skin-to-skin contact described issues of not enough space and time, and women’s families wanting to hold and see the baby immediately. In another example, while maternity staff agreed that routinely augmenting labour with oxytocin was harmful, it remained that midwives observed but had not yet been able to change this practice.

At the same time, in some of the hospitals with midwives without mentors, maternity staff indicated that midwives were improving the quality of care and practicing independently. Interventions mentioned included partograph use, companionship, birth position options, skin-to-skin contact, post-partum management (including family planning), breastfeeding, managing obstetric emergencies and using equipment for newborn resuscitation. It was shared that clinical exchange occurs, in which midwives shared their knowledge with the nurses, while nurses shared their expertise in other areas with midwives. In addition, midwives expressed a sense of competence in their roles and that they provide a higher quality of care than nurses. They also spoke about their ability and desire to treat emergency obstetric cases.

In spite of these shifts, maternity staff also expressed resistance to midwives working as autonomous care providers. For example, managers spoke of nurses’ competition with midwives, explaining that nurses felt that midwives were taking the nurses’ work, and that this led to nurses voicing that midwives were incapable of stepping into their roles as lead maternity care providers.

*“the nurses used to do the delivery care, but after the introduction of midwives the nurses cannot accept the midwives. That’s why the nurses believe that the midwives are not capable regarding knowledge and skill.”*

- Hospital Manager 1, midwives

An added element in this dynamic, shared by a doctor and hospital manager, was that nurses accept unofficial fees when they attend to births, while midwives do not. Indeed, some nurses admitted to accepting under-the-table payments for their services, while at the same time trivializing the amount. As one doctor shared,

*“I think the controversy of this is that the midwifery service is the better idea, and they execute the service for free.”*

- Hospital Manager 1, midwives

It was shared by some of the managers that it was common for midwives to have restricted clinical autonomy in hospitals without mentors and to work as nurses’ assistants during births, or even be assigned to general wards while nurses performed births. Notably, in the area of obstetric emergencies, many staff expressed that they did not feel that the midwives were competent. Midwives themselves described the experience of their supervisors preventing them from treating emergencies:

*“I am confident about my ability to manage this [obstetric emergencies] but it may happen that my seniors are trying to avoid this.”*

- Midwife 1, midwives

Midwives described that treating emergencies without the support of doctors put them at risk. Doctors shared that their resistance to caring for obstetric emergencies was due to the possibility that a woman might die in their facility and wanting to avoid potential retaliation from the community. Midwives shared that if the nurse in charge decides not to treat a woman who presents with an emergency they did not have the power to go against her. In this regard, midwives expressed frustration that their managers and supervisors restricted their autonomy, limiting both their scope and voice.

#### Hospitals with midwives and facility mentorship

*We are happy with the improved care the midwives have brought! (Maternity staff).*

*We feel proud! (Midwives with mentors).*

Overall, in settings where facility mentoring was ongoing, respondents communicated a general sense that the availability and quality of care was improving. Doctors, nurses and midwives expressed comfort with the new quality care interventions and with providing emergency obstetric care. Managers explained that the mentors facilitated positive relationships between midwives and nurses, and supported enabling environments for midwives and improved quality care. In addition, maternity staff spoke about midwives providing quality care autonomously, and expressed that maternity wards now had the needed expert staff.

Nurses specifically talked about midwives’ specialized education and gave examples of midwives expanding services, including counselling and education for women, and promotion of vaginal birth over caesarean section. They also expressed that the ANC that midwives provide is “correct”. Nurses in mentored hospitals were less concerned about the midwives’ youth. They rather referred to them as being young but mature and “not inferior” in knowledge. Supervisors described feeling better about the care given by midwives as opposed to nurses, and expressed that midwives have more expertise. Nurses and managers also talked about the midwives motivating the nurses to make positive changes.

A nurse shared that,

*“before the midwives joined the facility, we were not familiar with these techniques. When we saw these practicing in front of our eyes, then we felt motivated to do the proper service.”*

- Nurse 2, midwives with mentors

Staff and managers at the mentored facilities were the most likely to state that they do manage obstetric emergencies and some shared that this was relatively new. Most of the non-midwife maternity staff talked about midwives providing initial stabilization of emergencies. When asked if she was capable of resuscitating an asphyxiated newborn, a nurse stated that nurses are not comfortable with the new Ambu bag, but that the midwives were,

*“no, I don’t, the midwives do. I do mouth-to-mouth. The Ambu bag is very new, so I am not comfortable with it.”*

- Nurse 1, midwives with mentors

In some cases, nurses and managers described concerns about midwives managing emergencies. In one focus group, nurses talked about women lacking confidence in midwives’ ability to perform an emergency intervention for first trimester bleeding. In another instance, an obstetrician talked about midwives not being experienced enough to manage PPH and eclampsia yet, reiterating that women want doctors to treat emergencies. Yet, these were minor comments when weighed against the more frequent messages about greater willingness to respond to emergencies and greater use of evidence-based practices. One example is that nurses spoke of respectful care, and doing what women want. Nurses also explicitly talked about companionship helping women feel comfortable. When maternity staff were asked what has helped them make changes to more evidence-based care in their units, they described both the introduction of the new midwives, and the importance of mentoring.

One respondent shared that,

*“It was both the midwives and the mentors who made changes to the delivery position, and [appropriate use of] oxytocin for delivery, and increasing ANC.”*

- Nursing Supervisor 1, midwives with mentors

### Mixed methods results

The quantitative and qualitative findings were looked at together and found to largely agree, with some distinctions. Overall, the greatest resistance to quality care was conveyed in hospitals without midwives, and the greatest use of quality care was found in hospitals with midwives and mentors. There was some disagreement, however, between qualitative comments and survey results with regard to managing obstetric emergencies. In the survey more than 50% of all staff reported that they provided initial stabilization for eclampsia, and close to 100% of hospitals with midwives reported providing initial stabilization for women who present with PPH. However, in the focus groups all talked of referring a women with obstetric emergencies. In addition, and not in line with the study’s broader findings, hospitals with midwives were generally less equipped with the necessary supplies and equipment for responding to obstetric emergencies than the other hospital types. In the survey, maternity staff in hospitals with midwives but no mentors also reported slightly less confidence and action around responding to cases of eclampsia than maternity staff in hospitals without.

Table 8 shows a summary of all key quantitative and qualitative findings.

**Table 8:** Summary of all key results, qualitative and quantitative

**Measure**	**Hospital category **		
	**No midwives**	**Midwives**	**Midwives + mentors**
**1)Number of facilities** ready for obstetric emergencies	3 of 7	1 of 6	4 of 6
**2) Percent of staff** who valued, felt capable of using, and used evidence-based care practices	68%	81%	92%
**3) Number of evidence-based care practices** used > 50% of the time	4 of 8	6 of 8	8 of 8
**4) Number of evidence-based practices** with significantly greater use than in facilities without midwives (fixed effect logistic regression)	N/A (reference group)	3 of 8	5 of 8
**5) Midwives’ competence**	Staff imagined that having midwives would improve service provision.	Among nurses and managers, some affirmed midwives’ contribution, while some felt they were too inexperienced to be autonomous. Midwives expressed having the capacity to do more but not being allowed (e.g., some were not allowed to deliver babies).	Managers affirmed midwives’ capacities and contribution and midwives stated they were proud to be midwives.
**6) Separate ANC corners**	Non-existent	Transitioning to staffing ANC corners with midwives	ANC corners consistently staffed by midwives
**7) Management of obstetric emergencies**	Nurses reported that they do not manage obstetric emergencies (commonly, patients were referred).	Midwives expressed confidence in managing obstetric emergencies but have limited autonomy so were not able to if another staff person decided to refer.	Midwives said they managed obstetric emergencies.
**8) Caring for the poor**	Not discussed	Managers discussed under-the-table fees that motivated nurses to perform deliveries.	Midwives expressed a commitment to caring for the poor and providing services free of charge.
**9) Midwives’ pride**	Not discussed	Not discussed	Midwives stated that they “are proud to be providing quality care to women”.

## Discussion

The objectives of this research were to determine if 1) introducing international standard midwives in rural sub-district hospitals in Bangladesh, both with and without mentoring, improved the availability and quality of maternal and newborn health care; and 2) to explore the experiences of the midwives, and the maternity staff and managers they joined. It was found that ICM-standard diploma prepared midwives were able to negotiate complex systems, address barriers, and improve care quality and availability.

The quantitative findings suggest that midwives alone—without mentors—may increase the likelihood of women receiving four of the eight evidence-based birthing practices examined. Greater use of evidence-based practices was observed in hospitals with mentors creating an enabling environment by assuaging nurses’ and doctors’ concerns about midwives’ competencies and navigating solutions to the resistance posed. Some minor exceptions to the continuum were also noted. For example, while mentored hospitals performed well in readiness for obstetric emergencies, hospitals with only midwives underperformed in this area. In addition, the practices of companionship during labour and birth and AMSTL were routine in all hospitals, indicating that they are common even without midwives. Finally, in the survey, providers’ agreement with the value of evidence-based care was largely homogeneous, apart from delayed cord clamping and non-supine labour, the results for which did follow the continuum.

Three key observations were drawn from the qualitative analysis. First, it was noted that resistance to adoption of evidence-based care, including emergency care, was prevalent across hospitals. Habitual patterns of care not in alignment with recommendations were observed to be deeply ingrained. Second, the differences observed between the hospitals indicated that the presence of midwives lessened maternity staff and managers resistance to change, and that the least resistance occurred when mentors were present. Facility mentoring thus potentiated midwives’ employment of evidence-based antenatal and birth care, particularly in areas where complex changes were needed. Third, that midwives expressed pride in their roles, and an explicit motivation to serve the poor, indicates the possibility of broader social and economic repercussions of quality maternity care.

Together, the quantitative and qualitative findings were examined against the WHO health systems building blocks comprising leadership and governance, service delivery, financing, information systems, workforce, and access to essential medicines [29]. The results support that most of the health system building blocks were strengthened by the introduction of midwives, and further with mentors. The findings were also looked at in regard to the influence that quality maternity care may have on women’s perceptions of themselves, how others in their community see and treat them, and their financial solvency. In the following sections, we discuss these overall observations in greater detail.

The limitations of this study included insufficient data on the management of obstetric emergencies, possible loss of subtleties in the translation process, and that the small number of hospitals within each hospital type resulted in a loss of power in the mixed-effect regression models. Facilities did not sufficiently record obstetric emergencies coming from the community, as many were transferred before admission. This meant that the findings on this topic were limited to statements made by the midwives, maternity staff and managers. Translation was conducted by professional translators who did not have medical training. However, the translators did not have perfect fluency in English. This may have resulted in some nuances being lost in translation during interviews and in the transcriptions. In addition, although most aspects of both the midwives and the facilities in general were standard, some potential confounders such as the number of staff out on leave were not collected. Finally, the focus groups and interviews were short as they were carried out with working managers and health care staff this may have led to less depth of exploration.

### Resistance to change

Resistance to changes in healthcare delivery is generally motivated by a desire for control, entrenched habits, the perception that change would increase workload, and/or patient demand for existing practices [30]. For example, Alenchery et al. (2018) found that staff in India expressed resistance toward immediate skin-to-skin contact due to a perceived increased demand on their time [31]. Likewise, Payne et al. (2021) found resistance to delayed cord clamping in a multi-country study, despite the availability of both guidelines and mentorship due to entrenched habits [32]. In the present study, nurses and managers expressed resistance to adopting evidence-based practices, attributing their resistance to a lack of familiarity with the practices, inadequate time to perform them, and women’s preference for the status quo.

### Midwives and quality

Hospitals with globally standard midwives were observed to perform more WHO recommended quality interventions than those without midwives. The dramatic improvement for some of these interventions just with the introduction of midwives is remarkable. These findings contribute new knowledge to the field, as attribution to professional midwives for their roles in expanding both availability and quality of care in LMICs is still emerging. While many countries have had success introducing midwives as part of a package to improve maternal health, the research has not been able to zoom in on midwives, and specifically link them with transitioning to WHO recommended quality standards [6]. This study shows quality was improved in hospitals with midwives which is an area often recalcitrant to change. It also shows where resistance was too complex for midwives alone to institute a practice change, and where additional support to establish an enabling environment for change was needed.

### Facility mentoring was associated with complex change in routine and emergency obstetric care

While we acknowledge these successes, gaps in enabling environments for midwives in hospitals posed barriers. The ICM defines the enabling environment for midwives as one that, “supports the infrastructure, profession, and system-level integration needed for midwives to effectively practice their full scope of work”. It includes aspects of gender equality, infrastructure, professional status and agency, and system-level integration [33]. Barriers to enabling environments for midwives are common globally and were anticipated in this research [34–37].

Facilities with mentors had improved use of ANC cards, partograph, and upright positions for birth. Low use of these interventions is found in the literature from other LMICs. Both in Africa and Asia, including in Bangladesh, there are gaps in the use of these interventions [38]. Both partograph and ANC cards need to be acquired, and some learning curve [35]. Upright positioning for birth is facilitated by a squatting chair and requires countering the nearly universally supported paradigm within many health systems in LMICs in which birthing tables, which largely mandate supine birthing, are ubiquitous [39]. Research from Tanzania found that women used supine positions because their nurse-midwives guided them to, and nurse-midwives used them because they believed that it was the universally accepted position [40].

It is likely that mentors were able to successfully address complex barriers due to their status in the social hierarchy. As doctors themselves, they were more listened to by hospital managers, doctors and nurses. Their impact was thus largely derived from the combination of their social positioning as doctors, their knowledge of midwives’ scope of practice and evidence-based care, and their own scope of work around facilitating enabling environments for midwives.

Most of the existing literature only hints at a role for mentors focused on enabling environments, and tends to examine mentorship focused on capacity building. Only two articles published within the past 10 years described relationship and/or team building as part of mentors’ roles. One was a scoping review of mentorship interventions in LMICs aimed at improving the quality of primary health care. Four studies were included in the review, covering research in Rwanda, Afghanistan, Jordan and Botswana. Both relationship building and communication skills were identified as key elements of successful mentorship programs. The review specifically highlighted that mentorship plays a role in shifting power dynamics within social hierarchies in healthcare settings. It characterized this shift as being from didactic supervision to power-sharing [41]. In an article describing a nurse-led mentorship programme in India, rapport with managers and a team building approach including regular meetings were factors contributing to success [42]. The Bangladeshi mentors’ rapport building with hospital leadership and senior clinical staff, and their involvement of all relevant maternity care actors is in line with the principles that helped make the India intervention successful. Given gaps in literature documenting effective implementation of enabling environments for midwives in LMIC, this study makes important headway into offering a successful model.

The entrenched systemic barriers to providing emergency obstetric care were described in all facility types, but less so in those with mentorship. In this research, nurses, managers, and doctors described referring women facing critical emergencies to higher-level facilities without treatment. Midwives described their supervisors preventing them from providing emergency care. There is literature discussing emergency obstetric care refusal in Bangladesh, and in other countries [43, 44]. There is also documentation of patients in Kenya being admitted to higher-level hospitals having not received the needed care from rural referring hospitals [45]. We did not find research on midwives being restricted from providing emergency care, although the State of the World’s Midwifery 2021 report does describe countries with policies limiting midwives in certain emergency interventions [46]. These findings add to the global understanding of contributors to maternal death.

### Professional midwives contribute to a stronger health system

**Fig. 3:**
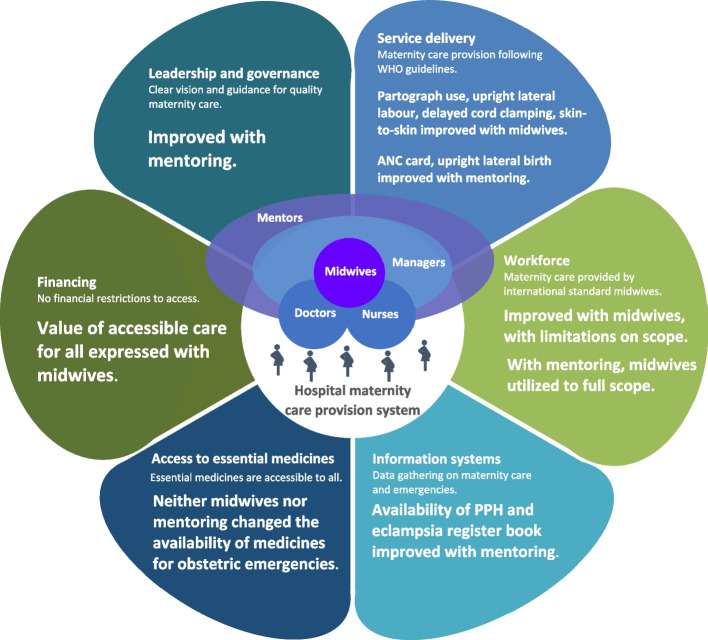
Alignment of findings with key health system components

The research found that the introduction of midwives contributed to the strengthening of nearly all of the health systems building blocks. Shown in Fig. 3, mentorship helped to align hospital managers’ endorsement of care practices with those backed by evidence, thus strengthening the leadership and governance of maternity care at the hospital level. The quality service delivery was associated with midwives’ deployment. Midwives themselves are the workforce providing maternity care and mentors facilitated using midwives utilized to their full scope. Greater availability of PPH and eclampsia register books in settings with midwives and mentors indicates a strengthened information system to track obstetric emergencies. Where there were midwives, more respondents expressed the value of accessible care for all, an indication of the role that midwives may play in making maternity care more equitable. Access to medicines for obstetric emergencies was not significantly different between facility types.

### Pride, quality, and equitable care

Midwives felt proud. This finding emerged without solicitation and was iterated with conviction. The profundity of this in a context in which taking care of women in labour has been considered dirty [47] has the potential for far reaching impact. Pride among healthcare providers has been described as an intrinsic motivator that improves healthcare provision [48]. Literature from high-income countries demonstrates that when midwives are enabled to practice their full scope, their pride improves, and so does care quality, even in stressful environments [49]. Notably, there is the possibility that being provided with quality maternity care may shift how women themselves, and their communities, consider women's worth [50, 51]. Furthermore, if poor women have access to quality maternity care, there may be less likelihood of catastrophic health expenditure [52]. Included in quality care is upholding rights of the most vulnerable. It may be that the ICM inclusion of quality care provision and the rights of women in their competencies coupled with enabling work environment, has the potential to instil pride. Perhaps because these midwives were adequately educated in the importance of human rights, and enabled, they felt more pride than lesser-educated providers. While this does not prove that adhering to a global standard of midwifery education improves maternal healthcare rights, it does give strength to the contention.

## Conclusions

This study’s findings support that ICM standard midwives can be a catalyst for change in the quality and availability of maternity care. It is one of the first studies to clearly find an association between midwives in an LMIC setting and better-quality care. Enabling environments after midwives’ deployment were crucial. With weaker enabling environments (i.e., without mentors) midwives improved quality, but greater quality improvement occurred with mentorship. In addition, managing critical patients improved with mentorship, but substantial gaps remained. Though this study was not designed for generalizability, the sample size is notable. The findings from this research can thus inform governments to create globally standard midwife cadres distinct from nurses, distinct midwife posts, and enabling environments for midwifery. Future research to refine the essential components of enabling environments for midwives, as well as mentorship, could stem from this study.

## Supplementary Information

**Additional file 1:Table S1.** Quotations and codes contributing to the theme “resistance to change”.

## Data Availability

The datasets analysed during the current study are available from the corresponding author on reasonable request.
