# Supplementary material for: The impact of professional midwives and mentoring on the quality and availability of maternity care in government sub-district hospitals in Bangladesh: a mixed-methods observational study
Source: BMC Pregnancy Childbirth. 2022 Nov 8;22:827. doi: 10.1186/s12884-022-05096-x (PMC9644636; doi:10.1186/s12884-022-05096-x)
Supplement: Supplementary file 1 — Additional file 1:Table S1. Quotations and codes contributing to the theme “resistance to change”. [file 12884_2022_5096_MOESM1_ESM.zip › 12884_2022_5096_MOESM1_ESM.zip/FGD Guideline_ Midwives_ESM.docx]

Key Topic Areas: For Midwives

**The researcher will ask the participant for the following introductory details:**

1. Confirm relevant personal contact details
2. Highest level of education
3. Number of years spent working as a midwife
4. Did the facility receive mentorship?

**Researcher will ask the staff to talk about their activities as an emergency/maternity staff:**

1. Has your hospital made any changes to improve maternal health care recently?
2. If so, can you describe what?
3. If so, what motivated these changes?
4. What helps you implement changes?
5. What helps you to learn a new clinical practice?
6. How do you feel about being a Diploma midwives
7. What changed since you came to this facility?
8. How do you feel about providing ANC
9. How do you feel about providing maternity care
10. Did your facility have a Save the Children midwifery led-care (SCI) mentorship program?
11. How do you feel about the Save the Children midwifery led-care (SCI) mentorship program?
12. What changed with Save the Children midwifery led-care (SCI) mentorship?
13. What do you do if a girl or woman comes to the Emergency Room/ Maternity with PPH/ eclampsia?
14. Do you know how to provide initial stabilization of PPH and eclampsia?
15. Which type of professional manages a woman with an obstetrical emergency first?
16. Describe how you monitor labour progress and foetal wellbeing?
17. Do you know how to use a partograph?
18. How do you feel about women in labor having a companion with them?
19. How do you feel if a woman in labor or during delivery is not in supine or lithotomy position?
20. When do you cut the cord?
21. How do you feel about delayed cord clamping?
22. What do you think about immediate skin-to-skin contact for one hour?
23. Do they feel you have the skills and information you need to use skin-to-skin contact?
    1. If not, what more would you need in order to feel capable?
24. Do you use skin-to-skin contact?
    1. What are the reasons for not using it?
    2. What is your experience of using it?
