# Supplementary material for: The impact of professional midwives and mentoring on the quality and availability of maternity care in government sub-district hospitals in Bangladesh: a mixed-methods observational study
Source: BMC Pregnancy Childbirth. 2022 Nov 8;22:827. doi: 10.1186/s12884-022-05096-x (PMC9644636; doi:10.1186/s12884-022-05096-x)
Supplement: Supplementary file 1 — Additional file 1:Table S1. Quotations and codes contributing to the theme “resistance to change”. [file 12884_2022_5096_MOESM1_ESM.zip › 12884_2022_5096_MOESM1_ESM.zip/Fixed effect regression_without covariates_ESM.docx]

| *Dependent variable*: | | | | | | | | |
| --- | --- | --- | --- | --- | --- | --- | --- | --- |
|  | **ANC Card** | **Partograph is used** | **Upright lateral labour** | **Companion present** | **Delayed cord clamping** | **Skin-to-skin contact (1hr)** | **Active management of the third stage of labour** | **Upright lateral birth** |
| **Intercept** | 1.08   \| (0.76, 1.55) \| \| --- \| | 0.17^**^  (0.08, 0.35) | 1.67  (0.97, 2.86) | 55^**^   \| (7.61, 397.44) \| \| --- \| | 0.13^**^  (0.05, 0.3) | 0.15^**^  (0.07, 0.34) | 16.67^**^  (5.2, 53.43) | 0.35^**^  (0.19, 0.66) |
| **Midwives without mentors†** | 0.74   \| \| (0.46, 1.21) \| \| --- \| \| \| --- \| --- \| | 4.84^*^  (3, 19.43) | 9.6^**^   \| (2.65, 34.73) \| \| --- \| | 0.91  (0.06, 14.92) | 56.14^**^  (16.83, 187.29) | 102.95^**^   \| (25.08, 422.64) \| \| --- \| | 2.94   \| (0.3, 29.24) \| \| --- \| | 2.16  (0.93, 5) |
| **Midwives with mentors†** | 4.72^**^   \| (2.83, 7.86) \|  \| \| --- \| --- \| | 174^**^  (35.27, 858.38) | 11.6^**^  (3.22, 41.73) | \| 4.22e+07 \| \| --- \|   (0e+00, $\infty$) | 438.67^**^   \| (51, 3.77e+03) \| \| --- \| | 88.71^**^   \| (24.42, 322.24) \| \| --- \| | 5.13e+07   \| (0e+00, $\infty$) \| \| --- \| | 18.14^**^ (6.83, 48.21) |
| **Number of observations** | 472 | 166 | 168 | 169 | 159 | 161 | 164 | 160 |
| Note: ⋆p<0.05; ⋆⋆p<0.00625 (Bonferrroni-adjusted alpha)  **†**Reference category: no midwives | | | | | | | | |

**Odds ratios and 95% confidence intervals for fixed-effect logistic regression models** **(no co-variates)**
