# Supplementary material for: The impact of professional midwives and mentoring on the quality and availability of maternity care in government sub-district hospitals in Bangladesh: a mixed-methods observational study
Source: BMC Pregnancy Childbirth. 2022 Nov 8;22:827. doi: 10.1186/s12884-022-05096-x (PMC9644636; doi:10.1186/s12884-022-05096-x)
Supplement: Supplementary file 1 — Additional file 1:Table S1. Quotations and codes contributing to the theme “resistance to change”. [file 12884_2022_5096_MOESM1_ESM.zip › 12884_2022_5096_MOESM1_ESM.zip/IDI_Guideline_ESM.docx]

**The researcher will ask the participant for the following introductory details:**

1. Confirm relevant personal contact details

2. Ask for the following basic demographic information:

gender; age; doctor, nurse, midwife

3. Highest level of education

4. Number of years spent working as a manager

6. Does the facility have professional midwives?

7. Did the facility receive mentorship?

**Researcher will ask the manager the following questions:**

**For all:**

8. Has your hospital made any changes to improve maternal health care recently?

9. If so, can you describe what?

10. If so, what motivated these changes?

11. What helps you implement changes?

12. What is your opinion about Diploma midwives?

13. How do you feel about having Diploma midwives working at your facility?

14. Do you have Diploma midwives working at your facility?

15. Does your facility provide ANC if so who provides it?

16. Does your facility provide evidence based routine maternity care? If so who provides it?

17.How does this facility respond when a woman comes to the ER/Maternity with PPH/ eclampsia?

18. If this facility treats the women what type of professional manages a woman with an emergency?

**These question will be asked If there are midwives posted at the facility:**

19. Have there been any changes since midwives were deployed to your facility? If so what?

20. What are the challenges of having midwives deployed to your facility ?

21. What are the benefits of having midwives deployed to your facility?

22. Do you feel like midwives are able to practice as midwives in your facility? If they are not can you describe how and in what way they are limited?

23. How do you feel about Diploma midwives providing independent ANC?

24. Do midwives provide independent ANC at your facility?

25. How do you feel about Diploma midwives providing maternity care including emergency care?

26. Has anything changed in ANC or maternity care provision including response to OB emergencies since midwives were posted?

27. Please describe any changes to me?

28. Did your facility have a mentorship program?

**These questions will be asked If there was mentorship at the facility:**

29. How do you feel about the mentorship program?

30. What were the challenges for the mentorship program?

31. Did anything change as a result of the mentorship program?

32. If yes can you describe those changes?
