# Supplementary material for: The impact of professional midwives and mentoring on the quality and availability of maternity care in government sub-district hospitals in Bangladesh: a mixed-methods observational study
Source: BMC Pregnancy Childbirth. 2022 Nov 8;22:827. doi: 10.1186/s12884-022-05096-x (PMC9644636; doi:10.1186/s12884-022-05096-x)
Supplement: Supplementary file 1 — Additional file 1:Table S1. Quotations and codes contributing to the theme “resistance to change”. [file 12884_2022_5096_MOESM1_ESM.zip › 12884_2022_5096_MOESM1_ESM.zip/Mixed effect regression_ESM.docx]

**Odds ratios and 95% confidence intervals for mixed-effect logistic regression models**

| *Dependent variable*: | | | | | | | | |
| --- | --- | --- | --- | --- | --- | --- | --- | --- |
|  | **ANC Card** | **Partograph is used** | **Upright lateral labour** | **Companion present** | **Delayed cord clamping** | **Skin-to-skin contact (1hr)** | **Active management of the third stage of labour** | **Upright lateral birth** |
| **Intercept** | 0.9 (0.09-9.08) | 0.01  (0.00-1.34) | 1.85 (0.68-5.02) | 55.00** (7.61-397.45) | 0.08**  (0.02-0.38) | 0.04*  (0.00-0.85) | 17.30**  (5.19-57.75) | 0.33 (0.03-3.46) |
| **Midwives without mentors†** | 0.37 (0.01-12.67) | 295.58 (0.22-4.03e+05) | 12.41* (1.96-78.56) | 0.91  (0.06-14.92) | 139.24** (11.55-1,678.31) | 2,999.12* (7.06-1.27e+06) | 2.94  (0.28-31.10) | 1.36 (0.04-47.59) |
| **Midwives with mentors†** | 13.67 (0.49-379.26) | 1.05e+05* (14.03-7.86e+08) | 15.17** (2.47-93.24) | 4.11E+13 (0.00-$\infty$) | 1,095.66** (48.76-2.46e+04) | 3,086.90* (6.76-1.41e+06) | 1.39E+14  (0.00-$\infty$) | 126.58* (2.49-6,431.42) |
| **Number of observations** | 472 | 166 | 168 | 169 | 159 | 161 | 164 | 160 |
| Note: ⋆p<0.05; ⋆⋆p<0.00625 (Bonferroni-adjusted alpha)  †Reference category: no midwives | | | | | | | | |
