# Supplementary material for: The impact of professional midwives and mentoring on the quality and availability of maternity care in government sub-district hospitals in Bangladesh: a mixed-methods observational study
Source: BMC Pregnancy Childbirth. 2022 Nov 8;22:827. doi: 10.1186/s12884-022-05096-x (PMC9644636; doi:10.1186/s12884-022-05096-x)
Supplement: Supplementary file 1 — Additional file 1:Table S1. Quotations and codes contributing to the theme “resistance to change”. [file 12884_2022_5096_MOESM1_ESM.zip › 12884_2022_5096_MOESM1_ESM.zip/Quant Form 1_Facility Observation Tool_ESM.docx]

**Please fill up only one Form for every facility.**

| **No.1** | **Address and identification information** | | | | | |
| --- | --- | --- | --- | --- | --- | --- |
| **1.1** | **Name of the Facility** |  |  | | | |
| **1.2** | **Upazila** |  |  | **1.3** | **Zila** |  |
| **1.4** | **Data collector's Name** |  |  | | | |
| **1.5** | **Date of Visit** |  | **\|___\|___\| : \|___\|___\| : \|___\|___\|___\|___\| (dd/mm/yyyy)** | | | |
|  |  | | | | | |

***Please circle correctly, which is observeed.***

| **No.** | **Variable** | **Yes** | **No** |
| --- | --- | --- | --- |
| **1** | **Oxytocin available in the emergency room** | **Y** | **N** |
| **2** | **Oxytocin available in the delivery room** | **Y** | **N** |
| **3** | **MgSO4 available in the emergency room** | **Y** | **N** |
| **4** | **MgSo4 available in the delivery room** | **Y** | **N** |
| **5** | **Newborn resuscitation Area with ambu bag in the delivery room** | **Y** | **N** |
| **6** | **Separate ANC corner** | **Y** | **N** |
| **7** | **Diploma midwife staffing the ANC corner** | **Y** | **N** |
| **8** | **Midwives staffing the maternity area** | **Y** | **N** |
| **9** | **Register book with midwife identification being used for deliveries** | **Y** | **N** |
| **10** | **Register book for PPH and eclampsia** | **Y** | **N** |
| **No.** | **Please collect the information from register book.** |  | |
| **11** | **Number of deliveries performed by midwives in the last six months** |  | |
| **12** | **Number of PPH cases in the last six months** |  | |
| **13** | **Number of eclampsia cases in the last six months** |  | |
|  |  |  | |
| **Before leaving the observational place, carefully check whether all answes of the questions have properly recorded.** | | | |

| ___________________________  (Signature of Data collector's)  Date:____/____/______ | ___________________________  (Signature of Supervisor's)  Date:____/____/______ |
| --- | --- |
