# Supplementary material for: The impact of professional midwives and mentoring on the quality and availability of maternity care in government sub-district hospitals in Bangladesh: a mixed-methods observational study
Source: BMC Pregnancy Childbirth. 2022 Nov 8;22:827. doi: 10.1186/s12884-022-05096-x (PMC9644636; doi:10.1186/s12884-022-05096-x)
Supplement: Supplementary file 1 — Additional file 1:Table S1. Quotations and codes contributing to the theme “resistance to change”. [file 12884_2022_5096_MOESM1_ESM.zip › 12884_2022_5096_MOESM1_ESM.zip/Quant Form 2_Clinical Observation Tool_ESM.docx]

Please fill up one sheet for one women.

| **No.1** | **Address and identification information** | | | | | |
| --- | --- | --- | --- | --- | --- | --- |
| **1.1** | **Name of the Facility** |  |  | | | |
| **1.2** | **Upazila** |  |  | **1.3** | **Zila** |  |
| **1.4** | **Data collector's Name** |  |  | | | |
| **1.5** | **Date of Visit** |  | **\|___\|___\| : \|___\|___\| : \|___\|___\|___\|___\| (dd/mm/yyyy)** | | | |
|  |  | | | | | |

***Operational Defination:***

**2.5 > Partograph:**

Partograph must be started within one hour or at 4cm. Fetal heart must be listen every 30 minutes & recorded. Dilatation must be filled correctly.

**2.6> Upright position:**

At least 90% non supine position.

**2.7>Companion:**

At least 90% of time companion present must be in delivery room.

**2.9> Skin to skin:**

On baby management before one hour.

***Please circle correctly, which is observeed.***

| **No.2** | **Variable** | **Yes** | **No** | **Not observed (NO)** |
| --- | --- | --- | --- | --- |
| **2.1** | **ANC card being used** | **Y** | **N** | **NO** |
| **2.2** | **Management of PPH observed** | **Y** | **N** | **NO** |
| **2.3** | **Management of Eclampsia observed** | **Y** | **N** | **NO** |
| **2.4** | **Management of Newborn asphyxia observed** | **Y** | **N** | **NO** |
| **2.5** | **Partograph is used during labour** | **Y** | **N** | **NO** |
| **2.6** | **Upright or lateral positions for labour/delivery** | **Y** | **N** | **NO** |
| **2.7** | **Companion present during labour/delivery** | **Y** | **N** | **NO** |
| **2.8** | **Delayed cord clamping until no pulse** | **Y** | **N** | **NO** |
| **2.9** | **Baby placed skin-to-skin immediately after birth and mother and baby were covered together in the first hour after delivery** | **Y** | **N** | **NO** |
| **2.10** | **Active management of 3^rd^ stage of labour** | **Y** | **N** | **NO** |
|  |  | | | |

| ___________________________  (Signature of Data collector's)  Date:____/____/______ | ___________________________  (Signature of Supervisor's)  Date:____/____/______ |
| --- | --- |
