# Supplementary material for: The impact of professional midwives and mentoring on the quality and availability of maternity care in government sub-district hospitals in Bangladesh: a mixed-methods observational study
Source: BMC Pregnancy Childbirth. 2022 Nov 8;22:827. doi: 10.1186/s12884-022-05096-x (PMC9644636; doi:10.1186/s12884-022-05096-x)
Supplement: Supplementary file 1 — Additional file 1:Table S1. Quotations and codes contributing to the theme “resistance to change”. [file 12884_2022_5096_MOESM1_ESM.zip › 12884_2022_5096_MOESM1_ESM.zip/Quant Form 3_ Survey Tool_ESM.docx]

**Introducing professional midwives in government sub-district hospitals in Bangladesh:**

**impact on quality of maternal and newborn health services**

| **No.1** | **Address and identification information** | | | | | |
| --- | --- | --- | --- | --- | --- | --- |
| **1.1** | **Name of the Facility** |  |  | | | |
| **1.2** | **Upazila** |  |  | **1.3** | **Zila** |  |
| **1.4** | **Data collector's Name** |  |  | | | |
| **1.5** | **Date of Visit** |  | **\|___\|___\| : \|___\|___\| : \|___\|___\|___\|___\| (dd/mm/yyyy)** | | | |
|  |  | | | | | |

| **No.2** | **Variable** | **Emergency Staff** | **ANC, or maternity Staff** | **Managers Staff** |
| --- | --- | --- | --- | --- |
| **2.1** | **Provider Name** |  |  |  |
| **2.2** | **Provider Type** |  |  |  |
| **2.3** | **Years of experience** |  |  |  |

***Please circle correctly, which is mentioned.***

| **No.3** | **Variable** | **Yes** | **No** |
| --- | --- | --- | --- |
| **3.1** | **Does your facility have Diploma midwives?** | **Y** | **N** |
| **3.2** | **Did your facility participate in a mentorship program (Save the Children midwifery led-care )?** | **Y** | **N** |
| **3.3** | **Is there a separate ANC corner in your facility?** | **Y** | **N** |
| **3.4** | **Do you feel that a separate ANC corner is important?** | **Y** | **N** |
| **3.5** | **Do you feel capable of using a partograph when a woman is in labor?** | **Y** | **N** |
| **3.6** | **Do you use a partograph when a woman is in labor?** | **Y** | **N** |
| **3.7** | **Do you feel capable of using skin-to-skin contact after delivery for one hour?** | **Y** | **N** |
| **3.8** | **Do you practice skin-to-skin contact after delivery for one hour?** | **Y** | **N** |
| **3.9** | **Do you feel capable to provide intial care for patients with PPH?** | **Y** | **N** |
| **3.10** | **Do you personally provide intial care to patients with PPH?** | **Y** | **N** |
| **3.11** | **Do you feel capable to provide intial care for patients with eclampsia?** | **Y** | **N** |
|  | **Do you personally provide intial care to patients with eclampsia?** | **Y** | **N** |

| **No.4** | **Variable (Likert scale, 1-5)** | **Strongly agree** | **Agree** | **Neutral** | **Disagree** | **Strongly disagree** |
| --- | --- | --- | --- | --- | --- | --- |
| **4.1** | **The partograph is helpful?** | **1** | **2** | **3** | **4** | **5** |
| **4.2** | **Mothers having a companion present for labor and delivery is a good idea?** | **1** | **2** | **3** | **4** | **5** |
| **4.3** | **Delayed cord clamping is a good idea?** | **1** | **2** | **3** | **4** | **5** |
| **4.4** | **Non supine position is important for pregnant and labouring women?** | **1** | **2** | **3** | **4** | **5** |
| **4.5** | **Skin-to-skin contact for one hour after delivery is the best care for mother and baby?** | **1** | **2** | **3** | **4** | **5** |
| **4.6** | **Having Diploma midwives in the ANC and maternity area is the best care for mother and baby?** | **1** | **2** | **3** | **4** | **5** |
| **4.7** | **If your facility participated in the Save the children (SCI) mentorship, was it helpful?** | **1** | **2** | **3** | **4** | **5** |
| **4.8** | **Recent introduction of Diploma midwives is helpful?** | **1** | **2** | **3** | **4** | **5** |

**In the below table, please mark any recent changes and then tell us if the change was related to mentoring and or the introduction of Diploma midwives. If the change has not happened, please leave it blank.**

| **No.5** | **Please tell us if this statement is**  **true or false** | **True**  **or**  **False?** | **Changed with mentors** | **Changed with recently deployed midwives** | **Comment** |
| --- | --- | --- | --- | --- | --- |
| **5.1** | **Before we did not receive women with PPH and eclampsia, now we do.** |  |  |  |  |
| **5.2** | **Before we did not have a separate ANC corner, now we do.** |  |  |  |  |
| **5.3** | **Before we did not use an ANC card, now we do.** |  |  |  |  |
| **5.4** | **Before we did not use a partograph, now we do.** |  |  |  |  |
| **5.5** | **Before all women were supine, now they are upright or lateral.** |  |  |  |  |
| **5.6** | **Before we did not allow companions in the delivery room, now we do.** |  |  |  |  |
| **5.7** | **Before we did not encourage oral hydration, now we do.** |  |  |  |  |
| **5.8** | **Before we did routine episiotomy on all primips, now we only do for fetal distress.** |  |  |  |  |
| **5.9** | **Before we cut the cord immediately, now we do delayed cord clamping.** |  |  |  |  |
| **5.10** | **Before we only put the baby skin-to-skin for one minute, now we do for one hour.** |  |  |  |  |
|  | | | | | |
| **Before leaving the interview place, carefully check whether all answes of the questions have properly recorded.** | | | | | |

| ___________________________  (Signature of Data collector's)  Date:____/____/______ | ___________________________  (Signature of Supervisor's)  Date:____/____/______ |
| --- | --- |
