# Supplementary material for: The impact of professional midwives and mentoring on the quality and availability of maternity care in government sub-district hospitals in Bangladesh: a mixed-methods observational study
Source: BMC Pregnancy Childbirth. 2022 Nov 8;22:827. doi: 10.1186/s12884-022-05096-x (PMC9644636; doi:10.1186/s12884-022-05096-x)
Supplement: Supplementary file 1 — Additional file 1:Table S1. Quotations and codes contributing to the theme “resistance to change”. [file 12884_2022_5096_MOESM1_ESM.zip › 12884_2022_5096_MOESM1_ESM.zip/STROBE checklist_ESM.pdf]

STROBE checklist for cross-sectional studies

	Item No	Recommendation	X = done	Pg. # and notes
**Title and abstract**	1	a. Indicate the study's design with a commonly used term in the title or the abstract	X	pg 1
		b. Provide in the abstract an informative and balanced summary of what was done and what was found	X	pg 1
**Introduction**				
Background/ rationale	2	Explain the scientific background and rationale for the investigation	X	pg 2
Objectives	3	State specific objectives, including any prespecified hypotheses	X	pg 2
**Methods**				
Study design	4	Present key elements of study design early in the paper	X	pg 3
Setting	5	Describe the setting, locations, and relevant dates, including periods of recruitment, exposure, follow-up, and data collection	X	pg 4
Participants	6	Give the eligibility criteria, and the sources and methods of selection of participants	X	pg 5 and 7
Variables	7	Clearly define all outcomes, exposures, predictors, potential confounders, and effect modifiers. Give diagnostic criteria, if applicable	X	pg 5
Data sources/ measurement	8	For each variable of interest, give sources of data and details of methods of assessment (measurement). Describe comparability of assessment methods if there is more than one group	X	pg 5
Bias	9	Describe any efforts to address potential sources of bias	X	pg 5
Study size	10	Explain how the study size was arrived at	X	pg 5
Quantitative variables	11	Explain how quantitative variables were handled in the analyses. If applicable, describe which groupings were chosen and why	X	pg 5 and 6
Statistical methods	12	a. Describe all statistical methods, including those used to control for confounding	X	pg 5 and 6
		b. Describe any methods used to examine subgroups and interactions	X	pg 5 and 6
		c. Explain how missing data were addressed	X	pg 5 and 6
		d. If applicable, describe analytical methods taking account of sampling strategy	X	pg 5 and 6
**Results**				

Participants	13	a. Report numbers of individuals at each stage of study—eg numbers potentially eligible, examined for eligibility, confirmed eligible, included in the study, completing follow-up, and analysed	X	Non-participation was not tracked and the exact number of eligible participants for the survey and focus groups was not known. However, the numbers roughly match what would be expected given staffing numbers.
		b. Give reasons for non-participation at each stage	X	
		c. Consider use of a flow diagram	N/A	
Descriptive data	14	a. Give characteristics of study participants (eg demographic, clinical, social) and information on exposures and potential confounders	X	pg 9 and 10
		b. Indicate number of participants with missing data for each variab	X	Table 1 Number of observations
Outcome data	15	Report numbers of outcome events or summary measures	X	pg 10, 11 and 12
Main results	16	a. Give unadjusted estimates and, if applicable, confounder-adjusted estimates and their precision (eg, 95% confidence interval). Make clear which confounders were adjusted for and why they were included	X	pg 12
		b. Report category boundaries when continuous variables were cat	N/A	
		c. If relevant, consider translating estimates of relative risk into absolute risk for a meaningful time period	N/A	
Other analyses	17	Report other analyses done—eg analyses of subgroups and interactions, and sensitivity analyses	X	All analyses are reported
**Discussion**				
Key results	18	Summarise key results with reference to study objectives	X	pg 20 and 21
Limitations	19	Discuss limitations of the study, taking into account sources of potential bias or imprecision. Discuss both direction and magnitude of any potential bias	X	pg 21
Interpretation	20	Give a cautious overall interpretation of results considering objectives, limitations, multiplicity of analyses, results from similar studies, and other relevant evidence	X	pg 21
Generalisability	21	Discuss the generalisability (external validity) of the study results	X	pg 25
**Other information**				
Funding	22	Give the source of funding and the role of the funders for the present study and, if applicable, for the original study on which the present article is based	N/A	There were no funders for this study, it is research that led to a PhD
