# Supplementary material for: The impact of professional midwives and mentoring on the quality and availability of maternity care in government sub-district hospitals in Bangladesh: a mixed-methods observational study
Source: BMC Pregnancy Childbirth. 2022 Nov 8;22:827. doi: 10.1186/s12884-022-05096-x (PMC9644636; doi:10.1186/s12884-022-05096-x)
Supplement: Supplementary file 1 — Additional file 1:Table S1. Quotations and codes contributing to the theme “resistance to change”. [file 12884_2022_5096_MOESM1_ESM.zip › 12884_2022_5096_MOESM1_ESM.zip/Table S1_Sample coding process.docx]

**Table S1 Quotations and codes contributing to the theme “resistance to change”**

| **Example quotations** | **Codes** | **Theme** |
| --- | --- | --- |
| *“We don’t even know about these positions, and we are habituated to doing delivery in lithotomy position.”*  *—*Obstetrician, (midwives without mentor)  *“We are also habituated with lithotomy position”*  *—*Midwife 1, (midwives without mentor) | Habituated to old ways | Resistance to change |
| *“We don’t allow any companionship. We don’t appreciate this.”*  *—*Nurse 6, (no midwives)  *“One hour is not comfortable”*  *—*Nurse 3 (no midwives) | Not comfortable with certain new interventions |  |
| *“If they don’t hand over the baby immediately after the delivery to the party (family), they think that there might be some problem with the baby or the mother.”*  *—*Hospital manager 2 (midwives without mentor)  *“Patients always prefer lithotomy, that’s why we didn’t use that (upright positions).” - —*Nurse 5 (midwives without mentors) | Women and their families want the existing routines, they do not want change |  |
| *“I cannot mention that officially, but it happens, it is the motivation for nurses to want to perform the deliveries.”*  *–*Doctor 2, (midwives without mentors)  *“I think the controversy of this is that the midwifery service is the better idea, and they execute the service for free.”*  *—*Hospital Manger 1, (midwives without mentors) | Nurses receive tips for performing deliveries |  |
| *“We do many works. We have* *work pressure. We do other work, and there is a lack of manpower. We cannot give time to them.”*  *—*Nurse 3 (no midwife)  *“We don’t have enough time* *to do that”*  *—*Nurse 5 (midwives without mentor) | Not enough time |  |
| *”Most of the time they (midwives) only help.”*  *—*Obstetrician 1 (midwives without mentor)  *“Do they (the midwives) deliver? I see you only deliver, and they are just your helping hand.” The nurses then replied that “yes only we do the delivery.”*  *–*Doctor1 (midwives without mentor and nurses 1-7 (midwives without mentor) | Midwives as nurses’ helpers |  |
| *“A few days ago, we made a training session for the nurses for maintaining the partograph (a globally recognized tool to document labor progress and maternal and fetal health); we think that we need more training.”*  *—*Resident Medical Officer 1, (no midwives)  *“The nurses are not well trained; even we (the doctors) are also doing lithotomy.”*  *—*Obstetrician 1, (midwives without mentor) | Need more training |  |
| *“From the student life, we have learned only lithotomy position. We didn’t even watch any other position.*  —Midwife 2 (midwife without mentors)  *“If someone shows us in front of our eyes, then it will be easier to learn.”*  –Midwife 1 (midwife without mentors) | Never saw it done |  |
| *“The nurses used to do the delivery care, but after the introduction of midwives, the nurses cannot accept the midwives.”*  *—*Hospital manager 1, (midwives without mentors)  *“After the introduction of the midwifery service, the* *nurses working before in that particular department, delivery service, they have some problems; they cannot accept the midwives.”*  *—*Hospital Manager 1, (midwives without mentors) | Nurses cannot accept midwives/competition |  |
